# γδ T-cells in human malignancies: insights from single-cell studies and analytical considerations

**DOI:** 10.3389/fimmu.2024.1438962

**Published:** 2024-08-30

**Authors:** Jeremy Wee Kiat Ng, Alice Man Sze Cheung

**Affiliations:** ^1^ Department of Anatomical Pathology, Singapore General Hospital, Singapore, Singapore; ^2^ Department of Hematology, Singapore General Hospital, Singapore, Singapore; ^3^ SingHealth Duke-NUS Medicine Academic Clinical Program, Duke-NUS Medical School, Singapore, Singapore

**Keywords:** cancer, gamma delta (γδ) T cells, bioinformatics & computational biology, immunotherapy, cell therapy (CT)

## Abstract

γδ T-cells are a rare population of T-cells with both adaptive and innate-like properties. Despite their low prevalence, they have been found to be implicated various human diseases. γδ T-cell infiltration has been associated with improved clinical outcomes in solid cancers, prompting renewed interest in understanding their biology. To date, their biology remains elusive due to their low prevalence. The introduction of high-resolution single-cell sequencing has allowed various groups to characterize key effector subsets in various contexts, as well as begin to elucidate key regulatory mechanisms directing the differentiation and activity of these cells. In this review, we will review some of insights obtained from single-cell studies of γδ T-cells across various malignancies and highlight some important questions that remain unaddressed.

## Introduction

γδ T-cells are present at a low frequency of between 0.5 – 1.6% in the human peripheral blood while being found at higher abundance in tissues such as the liver and intestinal lining (1, 2). Despite this, they are involved in modulating immune response against a range of infections of both bacterial and viral origins (3, 4). Since the 90s, there has been also an emerging body of literature suggesting the involvement of these cells in leukemic and solid malignancies. The significance of these cells in human malignancies was subsequently highlighted by Gentles and colleagues, where they demonstrated using deconvolution of bulk RNA-seq that increased γδ T-cell infiltration was associated with improved prognosis across different disease indications (5). Since then, renewed interest in leveraging these cells for cancer immunotherapy has culminated in various clinical trials to determine their efficacy (6, 7). However, these clinical trials yielded mixed results, calling for more extensive characterization of these cells. Single cell approaches that allow high-resolution insights into these cells in various physiological and pathological settings have enabled these detailed characterizations. In this review, we focus on key single-cell studies of human γδ T-cells in the past decade, highlighting some of the significant findings of relevance in γδ T-cells based cancer immunotherapy.

## Challenges of scRNA-seq analysis in the context of γδ T-cell biology

There are various technical challenges in single-cell RNA-seq (scRNA-seq) with potential implications on the analysis and interpretation of results. One issue is zero-inflation, where an absence of reads mapping to a gene could be biological (no gene expression) or non-biological (e.g. drop-outs) in origin. Drop-outs have been shown to impact various downstream analyses such as clustering and differential gene expression (8). Various imputation methods have been proposed to recover the actual expression of genes with zeroes to reduce its impact on downstream analysis. A rigorous benchmark study demonstrating that improvements in results were obtained with SAVER (9) and NE (10, 11). While imputation can be used to recover the expression of genes, it can also lead to masking of signals from rare cell populations such as γδ T-cells in complex cell mixture. This is because “zero read”-gene imputation is performed by considering that gene’s expression observed in cells with similar transcriptomic profiles. The rarity of γδ T-cell could lead to the masking of their gene expression profiles by that of highly similar, yet more prevalent cell types such as CD8 T-cells and NK cells (12). Additionally, the presence of non-biological zeroes in scRNA-seq also complicates gene module scoring. Gene module scoring is a common approach used to inform cluster annotation. The most common gene module scoring approach is implemented in Seurat and uses the sum of expression of genes in the signature as the input for score calculation (13). Various other scoring schemes [reviewed by Zhang and colleagues (14)] are ranked based. However, regardless of whether scores are calculated by summing or ranking the expression of genes in a module, the presence of zeros can lead to an underestimation of pathway activity, problematizing cluster characterization.

Another challenge encountered in scRNA-seq analysis is cell clustering and annotation. A key requirement for optimal cell clustering is sufficient variation between the transcriptomic landscape of different cell types. However, this is not the case with γδ T-cells. Instead, γδ T-cells have transcriptomic landscapes similar to CD8 T-cells and NK cells (12). In a seminal study, Pizzolato and colleagues demonstrated that a high clustering resolution of 1.2 was required to differentiate γδ T-cells clustered among CD8 and NK cells (12). This resolution is higher than the default value of 0.8 used in the popular scRNA-seq analysis software Seurat (15). The increased clustering resolution comes at the expense of an increase in sub-divisions of cell types. In the study by Pizzolato and colleagues, they reported the subdivision of monocytes (from three to six clusters), B-cells (from one cluster to two clusters) and T-cells (from two clusters to eight clusters) (12). This increase in number of reported clusters poses significant challenges to cell annotation as researchers need to discern between noise and biologically meaningful differences between clusters.

## γδ TILs in human malignancies

**Figure 1:**
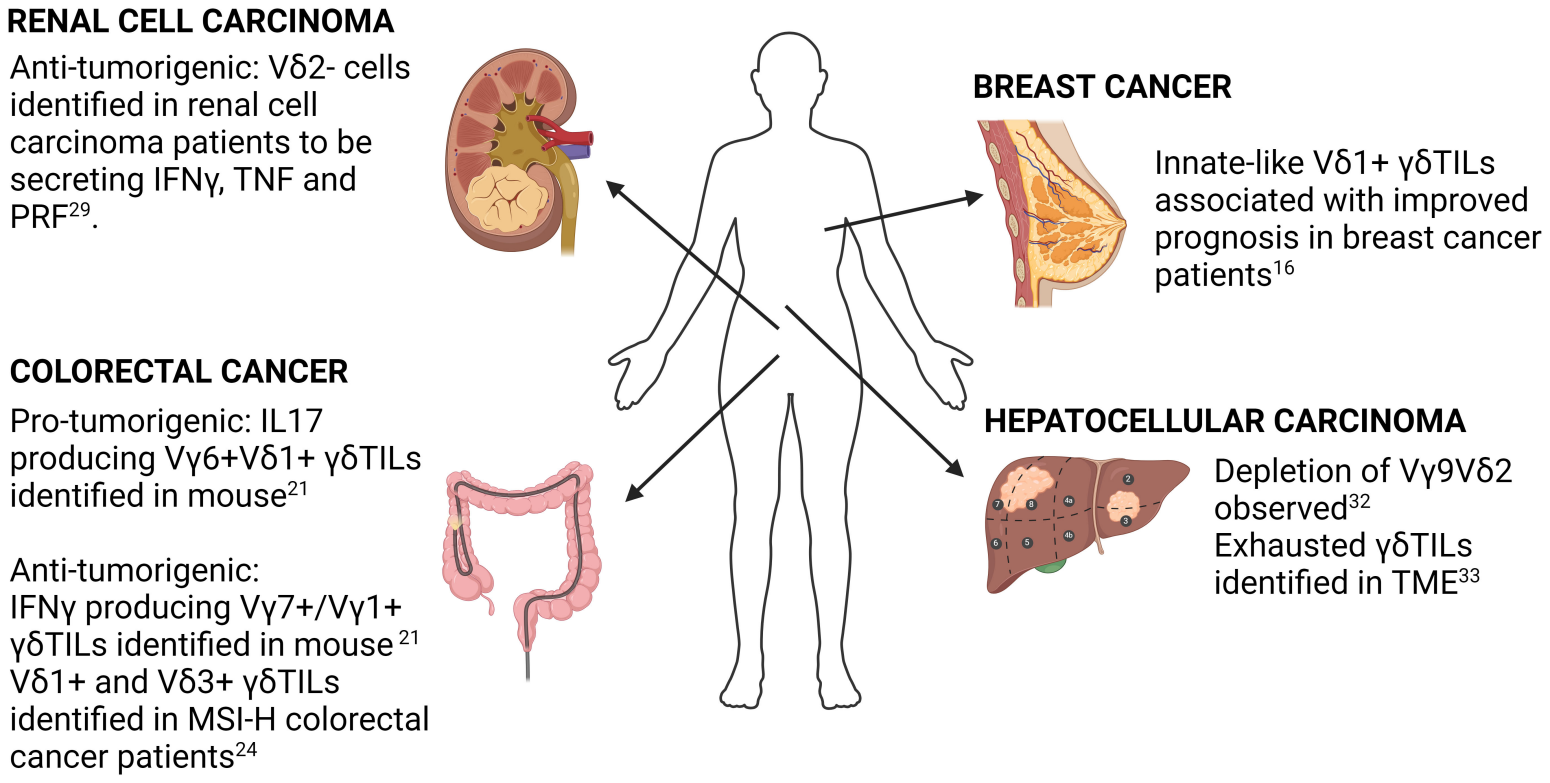
Summary of key findings in selected solid cancers. Key findings in γδ T-cell biology in breast cancer, colorectal cancer, hepatocellular carcinoma and renal cell carcinoma using both human and mouse samples are highlighted. Detailed description of each study is found in the accompanying text.

Having discussed some of the major technical challenges in the analysis of scRNA-seq data, we turn to critically review some of the key findings made in tumor-infiltrating γδ T-cells (γδTILs) characterized using scRNA-seq in the following 4 human malignancies (**Figure 1**).

### Breast cancer

scRNA-seq study of γδ TILs in the context of breast cancer was performed by Boufea and colleagues (16). Using unsupervised clustering of scRNA-seq from purified γδ T-cells, they identified three clusters of γδ TILs (T1, T2 & T3), with the gene signatures of T2 being the only one showing significant correlation with improved survival in TCGA breast cancer patients. Interestingly, the authors also noted that the T2 gene signature score inversely correlated with expression of αβT cell markers, cytolytic scores as well as breast cancer tumor mutation burden.

To deduce the origin of γδ TILs (tissue resident vs re-circulating), Boufea and colleagues used scID (17) to perform label transfer to compare circulating γδ T-cells with γδ TILs in breast cancer patients. Using this approach, they deduced that the clinically relevant T2 subset was of a tissue origin. Label transfer is a valuable approach that can be used to overcome the challenges of cluster annotation. It annotates cells by comparing the transcriptomic landscape of labelled cells (reference) with the unlabeled cells (query). A key requirement of this approach is the need for a comprehensive atlas of γδ T-cell effector types. Additionally, this approach does not preclude the possibility of cells acquiring a different effector subtype upon tumor infiltration and hence can only suggest an absence of shared γδ T effector subtypes between PB and the tumor microenvironment (TME). A more definitive approach to address this question is the use of paired single-cell T-cell receptor (TCR) sequencing (scTCR-seq) that enables clonotype tracking to show that there are no shared clones between the PB γδ T-cell and γδ TILs in the TME. However, paired scTCR/scRNA-seq can be prohibitively expensive for large number of cells. An attractive alternative is the use of 5’ scRNA-seq to infer TCR sequences. Various approaches have been developed to enable such analysis, including TRUST4 (18) and MiXCR (19). The value of this approach was demonstrated by Li and colleagues who demonstrated that the use of TCR sequence inferred from RNA-seq enabled the identification of γδ T-cells that had been previously annotated as CD8+ T-cell due to their transcriptomic profile. However, a potential limitation of such an approach is the inability to identify low frequency clonotypes. This was highlighted in the study by Peng and colleagues, who found that bulk RNA-seq based TCR inference can accurately identify abundant clonotypes but are limited in ability to identify low frequency clonotypes as well as perform TCR reconstruction in samples with low T-cell contents (20). Whether scRNA-seq based inference of TCR sequences suffers from the same limitations remains to be rigorously benchmarked.

### Colorectal cancer

γδ TILs have been extensively characterized in colorectal cancer (CRC), with both anti-tumorigenic and pro-tumorigenic functions identified for these cells. The first scRNA-seq study to support a pro-tumorigenic function of γδ TILs in CRC was from Reis and colleagues (21). They leveraged a tumor-adjacent normal study design to address the question of whether γδ T-cells in the TME were distinct from those in normal colonic tissue. They found that γδTILs had increased expression of genes associated with IL17 producers such as CD9 and LGAL3 whereas cells in the adjacent normal tissue had higher expression of cytotoxicity related genes and an enrichment of an IFNγ gene signature. This data was the first to show that transcriptionally distinct γδ T cell populations were spatially segregated within the same tissue organ and suggested that the tumor microenvironment might have an active involvement in γδ T cell functional reprogramming.

To further relate effector subtype to clonotype, the authors performed scTCR-seq in mouse models of CRC. They found that effector subtype was associated with Vγ gene usage, with a more clonally diverse Vγ7+/Vγ1+ subtype being IFNγ producing and the Vγ6+Vδ1+ had more pronounced clonal focusing that mapped to IL17 producing γδ T-cells. The authors used the total number of distinct clonotypes (defined using the CDR3 amino acid sequence) as a measure of clonotype diversity, which in turn was associated with clonal focusing. While intuitive to interpret and adequately capturing diversity, this metric does not provide information on clonotype evenness and can be influenced by under-sampling. Hence, while the overall claim of reduced diversity in the dominant clonotypes is sound, a more cautious interpretation of clonal focusing is warranted given the sensitivity of their metric to under-sampling. Instead, clonal focusing can be more robustly quantified using other ecology-based metrices, such as the Gini coefficient or Gini-Simpson index. One suitable metric that can be used is the Gini-Simpson index which was demonstrated to be less sensitive to low frequency clones and more robust to under-sampling (22). Another popular metric is the Gini coefficient that measures inequality, with a low Gini coefficient reflecting a lack of clonal focusing and a more innate-like mode of expansion (23).

On the other hand, a more pronounced anti-tumorigenic role of γδ TILs has been identified in CRC patients with microsatellite instability (MSI). The study by De Vries and colleagues (24) demonstrated that γδ TILs are the main effector of the sustained immune checkpoint blockage (ICB) response in MSI-high (MSI-H) patients displaying HLA-I defects (25). Deducing from the scRNA expression of TRDV gene alone (with a low threshold of 1), the authors concluded that Vδ1+ and Vδ3+ T cells were the more prevalent γδ T cell subsets in β2-microglobulin (B2M) deficient MSI-H CRC. The use of TRDV gene expression alone for identification of subtypes can lead to false negatives due to the zero-inflated nature of scRNA-seq data. A better approach for identifying which γδ T cell subtype at the single-cell level would be the use of TCR reconstruction or a more comprehensive gene panel such as that proposed by Pizzolato that considers the overall sequencing characteristics by performing both intra and inter-cell normalization. Nevertheless, the overall conclusion of De Vries study remains sound especially with the substantiation of additional functional validations.

The role of γδ T-cells in MSI-H CRC was further highlighted in the study from Harmon and colleagues (26). Comparing between MSS and MSI-H CRC, they found that a subset of γδ TILs with high cytotoxicity (characterized by expression of PRF1, GZMA, CCL5, ENO1, PKM and GNLY) was enriched in mismatch repair (MMR)-deficient CRC, whereas the less cytotoxic, PLZF^+^ wound-healing γδ TIL subset (with high expression of ZBTB16, AREG, TAGLN2 and CD44) was associated with MMR-proficient CRC (26). To account for non-biological sources of variation across different patients, the authors used Harmony for integration (27). Because Harmony uses a model-based approach for batch correction, all sources of variation must be provided. In practice, not all variables can be identified. Instead, other model-free approaches such as CCA for integration can be employed (15). Additionally, Harmony optimizes a maximum diversity function that penalizes clusters with low cell-origin diversity. This leads to a preference for clustering solutions that have high batch heterogeneity. A potential consequence of this is the loss of biologically meaningful patient-specific or subtype-specific effector cells. From the analysis of the combined dataset, the authors identified AREG as a key modulatory gene that exerts a pro-tumorigenic function. This finding is substantiated by wound healing assays showing that Vδ1+ cells producing AREG led to increased cell proliferation and migration.

Taken together, these studies not only confirmed the heterogenous nature of γδ TILs within CRC, but also highlighted their dynamic interactions with the TME that contribute to their functional diversity. Further work remains to be done to elucidate how the TME regulates functional diversity of γδ TILs in CRC.

### Renal cell carcinoma

The first signal suggesting a role of γδ TILs in renal cell carcinoma (RCC) was from a FACs study by Lee and colleagues (28). They found variable cell surface CD3 expression level among γδTILs. The CD3^lo^ population was found to be FAS+CD28+ indicating a chronic activated state. Interestingly, the CD3 expression levels was associated with TRδV usage, with CD3^lo^ cells being Vγ9δ1 whereas the CD3^hi^ cells were dominantly Vγ9δ2. Additionally, they found that the infiltrating Vγ9δ1 was biased towards a cytotoxic phenotype rather than cytokine production, indicated by an inability to secrete cytokines in response to phorbol myristate acetate or ionomycin.

The cytotoxic role of the Vγ9δ1 population in the RCC TME was corroborated in a later study by Rancan and colleagues (29). They found that tumor infiltrating γδ T-cells are mainly Vδ2- cells that were functionally heterogeneous. Consistent to earlier studies (30), Vδ2+ and Vδ2- had distinctive transcriptomes. They found that the Vδ2- cells population expressed higher levels of exhaustion markers PD1, TIGIT and TIM3. Interestingly, when cultured ex-vivo, the authors found that Vδ2- cells expressing high levels of these exhaustion markers were able to secrete comparable amounts of effector molecules such as IFNγ, TNF and PRF1. Additionally, the “exhausted” γδ T-cells were also able to retain cytotoxicity against RCC tumors. These findings challenge the conventional paradigm of T-cell exhaustion exemplified by αβ T-cells and raises the question of the extent to which effector subtypes are shared between αβ T-cells and γδ T-cells despite studies suggesting similarity in developmental trajectories (31). Further studies to identify γδ T-cell specific exhaustion markers is required.

### Hepatocellular carcinoma

Zakeri et al. had shown via a multi-parameter flow cytometry that the Vγ9Vδ2 subset was selectively depleted within hepatocellular carcinoma (HCC) (32). This does not appear to be due to a defect of Vγ9Vδ2 T-cells in homing to the liver as the authors demonstrated the ability of both Vδ1 and Vγ9Vδ2 T-cells to acquire a tissue-resident memory T cell (T_RM_) like phenotype characterized by expression of CD69/CD49A or CD69/CD103. Of note, γδ T cells with T_RM_ phenotype were also shown to display long term hepatic retention, arguing against the active egress of Vγ9Vδ2 T-cells into circulation. Functionally, T_RM_^+^ γδ T cells were found to favor towards cytokine production rather than being cytotoxic. Nevertheless, the specific roles of the different γδ T cell subsets in HCC remain poorly defined and awaits further investigations.

scRNA-seq to characterize human liver-associated γδ T-cells was performed by He and colleagues (33). They found that γδ T-cells from both healthy controls and HCC patients formed six clusters, with only one cluster (c4) originating from HCC patients. Functional analysis of the gene expression of this cluster found that γδ TILs had high expression of stress marker genes such as GADD45γ and GADD45β, the exhaustion marker gene LAG3 and cytotoxic genes such as NKG7, GNLY, GZMB and IFNγ. The combination of gene markers suggest that the γδ TILs are an exhausted but cytotoxic population within HCC tumors. This is in keeping with the findings by Rancan and colleagues in RCC (29). By combining trajectory analysis with RNA velocity analysis, they demonstrated that the developmental trajectory of γδ TILs was unidirectional, developing from a naïve state through various transitionary state before being irreversibly exhausted. Gene enrichment analysis also revealed that γδ TILs in HCC had extensive metabolic re-wiring with increased expression of genes related to glutamine metabolism. Whether the change in gene expression leads to changes in the metabolome remains unclear and awaits clarification in future studies.

Taken together, data from existing research on γδTILs confirm that exhausted γδ T-cells can be found in the TME of HCC patients. The results from He and colleagues suggest that exhaustion is driven by LAG3. However, whether ICB can be used to re-activate exhausted γδ T-cells in the TME remains unclear. Additionally, whether decreased TCR diversity in γδ TILs found in the HCC TME has prognostic or functional implications remains unclear.

## Normal human γδ T-cells – avenues for adoptive cellular therapies

Increasing our understanding of γδ T-cells in the tumor microenvironment is pivotal in enabling adoptive cell therapy (ACT). Here, we briefly review some of studies that will enable translation of γδ T-cell based ACT.

### Cord blood vs adult PB derived γδ T-cells

While peripheral blood (PB) serves as the conventional source of γδ T-cells, cord blood (CB), with extensive worldwide banking, offers a readily available alternative. However, γδ T-cells from both sources have been shown to differ significantly. A key difference between CB and PB γδ T-cells is the clonotype diversity observed in both sources. CB γδ T-cell repertoire has been reported to be more complex (34) with more extensive usage of TRδV1 chain. On the other hand, PB γδ T-cells are dominantly the Vδ2Vγ9+ subtype with more restricted clonotype diversity.

Relating to difference in TRδV usage, Tan et al. performed scRNA-seq comparing PB and CB γδ T-cells. To facilitate the analysis of marker genes to functionally characterize cell clusters, they applied unsupervised clustering based on the average expression of these marker genes in each cluster to identify gene modules that are associated with different biological processes. Using this approach, they found that neonatal γδ T-cells were distinctive from adult γδ T-cells. This approach of using co-expression to identify gene modules has been implemented in Monocle (35). Other than the use of co-expression, co-regulation by shared transcription factors can also be used to identify gene modules. The identification of genes under shared regulation is the basis for SCENIC (36), which leverages publicly available chromatin immunoprecipitation sequencing (ChIP-seq) datasets to score the activity level of regulons. Finally, another approach that can be used for functional characterization of cell clusters is the use of gene module scoring. A combination of functional characterization methods is often used for *ab-initio* cluster analysis to ensure accurate labelling of cell clusters which are reflective of effector subtypes in the absence of a reference cell atlas. Leveraging on a pan-immune cell dataset from developing the developing thymus, Tan and colleagues also suggested that IL17 producing γδ T-cells arise early in the embryonic thymus. However, the small number of γδ T-cells in the dataset did not allow analysis of how IL17 γδ T-cells could have developed throughout thymic development.

### *In vitro* expanded γδ T-cell products

Despite the well-established phosphoantigen stimulated expansion of Vγ9+Vδ2+ cells, there is a paucity in high resolution phenotypic and transcriptional characterization of these cells in *in vitro* expanded cell products. At the same time, development of protocols for human Vδ1+ cell expansion has been lagging, and in turn affecting the progress in understanding the mechanisms involved in regulating the activities of these cells. Our team has previously adopted a modified rapid expansion protocol (REP) to study the differential behaviors of the various CB derived γδ T cell subsets when subjected to the same stimulatory signals (ie: culture system). We found that upon culture stimulations, naïve CB γδ T cells adopted at least two majorly distinct developmental trajectory that reflects differential functional cell states (37). Developmental trajectories were inferred using Slingshot (38), which has been shown to be robust across different differentiation topologies (39). Although all γδ T cell subtypes were represented in the different cell states, we observed a much higher propensity for the Vδ2- subsets to acquire the cytotoxic cell phenotype compared to the Vδ2+ counterpart. Work remains to be done to identify critical gene regulatory programs that drive cell differentiation into each state.

## Analytical innovations can shed deeper insights into γδ T-cell biology

**Figure 2:**
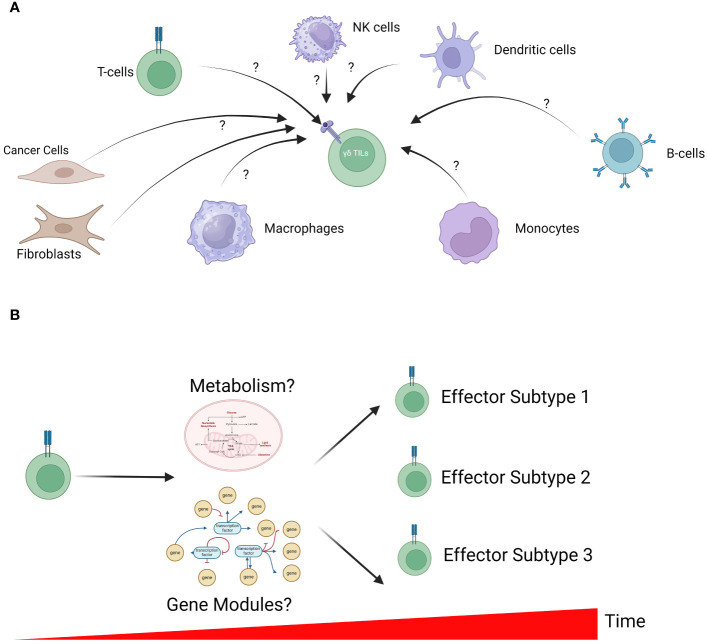
Unresolved questions in γδ T-cell biology. **(A)** The microenvironment of solid tumors is a complex admixture of various cell types, each with the potential to influence γδ TILs. However, how these cells function to regulate γδ TIL activation remains unclear. In-silico analysis to identify cell-cell interactions is a powerful approach to generate testable hypotheses for further validation in *in-vitro* studies. **(B)** γδ T-cell differentiation is a dynamic process that is tightly controlled by cell metabolism (including energy production and biosynthesis) and transcriptomic profiles. The interplay between metabolism, gene expression profile and γδ T-cell development remains largely unclear; however, further understanding can inform future manufacturing of these cells for therapy.

The increasing accessibility of scRNA-seq led to rapid innovation in analytical strategies that in turn shed deeper insights into the biology of γδ T-cells. Here, we highlight a few key avenues of future inquiries to further our understanding of γδ T-cells in human malignancies that is enabled by both established and emerging bioinformatics tools in other scRNA-seq studies (**Figure 2**). We also suggest some bioinformatics tools that have been developed for these specific analyses (**Table 1**).

**Table 1:** Analytical tools and approaches.

Tool	Function	Reference
scID	Label transfer using discriminant analysis of marker gene expression	Boufea et al (17)
Seurat	Dimension reduction, unsupervised clustering, transcriptome-based label transfer, multi-modal and integrative analysis of single-cell dataset	Hao et al (15)
scMRA	Label transfer using deep learning	Yuan M, Chen L, Deng M. (40)
graph-sc		Ciortan M, DeFrance M. (41)
Monocle3	Basic analysis as per Seurat, gene module inference, trajectory inference	Trapnell et al (35)
SCENIC	Inference of gene regulons	Aibar et al (36)
Slingshot	Trajectory inference	Street et al (38)
TRUST4	TCR sequence from 5’ gene expression	Song et al (18)
MiXCR		Bolotin et al (19)
scGate	Digital gating for identification of cell type	Andreatta M, Berenstein AJ, Carmona SJ. (42)
CellPhone DB	Inference of cell-cell communication	Efremova et al (43)
CellChat		Jin et al (44)
METAFlux	Inference of metabolic flux from scRNA-seq	Huang et al (45)

This table provides a non-exhaustive list of some of the tools that have been developed for the bioinformatics analysis of scRNA-seq.

While purifying γδ T-cells enable characterization of effector subtypes, these studies preclude the analysis of cell-cell communication between different cell types. Hence, a biologically relevant approach is to perform scRNA-seq on admixtures from the TME. A critical first step is to accurately label cell types present in the admixture. This remains a hurdle that has not been adequately addressed, as evidenced by the lack of γδ T-cells identified across various pan-immune cell atlases have been published (46, 47). The lack of γδ T-cells identified can be due to their low frequency and transcriptomic similarity to CD8 T-cells and NK-cells (12). There is a need for more sensitive approaches to identify rare populations of γδ T-cells. In this space, innovative signature-based and signature-free approaches have been developed. A drawback of signature-based approaches is the need for defined gene signatures identifying γδ T-cells. This remains an area of active research. However, an advantage of the use of signature-based approach is the interpretability of these models. This is exemplified by scGate (42), a digital gating approach used to classify cells in a manner reminiscent of flow-cytometry assisted cell sorting. On the other hand, signature-free approaches depend on the identification of suitable low-dimension embedding for label transfer. In this space, various deep learning-based approach for label transfer using graph neural networks (GNN) has been proposed (40, 41). A key advantage of the use of GNN-based approaches is their insensitivity to batch effects and the ability to handle incomplete reference dataset annotation due to the ability of GNNs to learn graph structures. Despite their power, the use of GNN-based approach for label transfer is limited by need for powerful graphic processing units (GPUs). The increasing accessibility of GPUs alongside the availability of additional annotated datasets will enable further advances and adoption of GNN-based approaches for label transfer. Another drawback of these approaches is the lack of interpretability of these models.

Studies have pointed to the role of cell-cell communication between immune cells and fibroblasts (48–50) and endothelial cells (51) have been suggested to regulate the immune tumor microenvironment. The identification of these crosstalk can be done using methods to identify cell-cell communication such as CellPhone DB (43) and CellChat (44). These methods utilize existing knowledge of ligand-receptor interactions and protein-protein interactions to infer potential cell-cell interactions. The low abundance of γδ T-cells in the TME could serve to limit the ability of these algorithms to detect meaningful cell-cell interaction in two ways. Firstly, the requirement for heterogenous cell mixtures without selection could lead to inability to identify γδ T cells in the mixture. As discussed previously, more sensitive methods to identify γδ T cells is crucial for enabling such integrative analyses. Additionally, the low abundance of γδ T-cells could hinder the identification of cell-cell communications between γδ T cells and other compartments due to the masking by more dominant cell communication networks between the major compartments to control false discovery rate. In this space, future work to improve sensitivity of these methods while controlling false discovery could be instrumental in understanding how tumor infiltration of γδ T-cells is regulated and improve patient selection of γδ T-cell based therapy.

The role of metabolism in regulating cell differentiation was elegantly demonstrated by Lopes and colleagues, who demonstrated that IL17 and IFNγ producers were metabolically distinct, with the former extensively using oxidative metabolism and the latter was exclusively glycolytic (52). Various studies in γδ TILs have also suggested a role of metabolic re-wiring, with an involvement of glutamine metabolism and AREG metabolism in HCC (33) and CRC γδTILs (26) respectively. These important role of metabolism in influencing γδ T-cell development is in keeping with other immune cell types as described in the growing body of literature in immunometabolism (53–57). While most studies in γδ T-cells have inferred metabolic rewiring based on the expression of key metabolic genes, being able to characterize metabolic fluxes at the single-cell level can be a powerful approach to enable the efficacy of *in-vitro* expanded γδ T-cells. Flux balance analysis, such as that implemented in METAFlux (45), has emerged as a computationally efficient approach to address this question in other contexts such as the metabolic landscape of tumors but has yet to be extended to the analysis of γδ T-cells in both physiological and pathological contexts.

Unlike the adaptive nature of αβ T-cells, γδ T-cells possess a mix of both adaptive-like and innate-like subpopulations with distinctive roles in disease control. Various studies have suggested that the innate-like γδ T-cells are critical to the antitumorigenic functions of γδ TILs (23, 24) in various cancers. However, what regulatory programs determine cell fate along the adaptive-innate axis remains unclear. Recently, our group characterized the transcriptomic landscape of the clonotypically diverse cord blood derived γδ T-cells (CBγδ T-cells) following *in-vitro* expansion using a time-course experimental design (37). Although there was clonotype-dependent bias in adaptive-like and innate-like expansion, clonotype-specific expansion was not observed in either compartment (37). The results point to the role of other yet-undetermined factors that determine whether γδ T-cells adopt a more adaptive-like or innate-like profile. Identifying both regulatory programs and biomarkers of innate-like γδ T-cells remains an important but unaddressed question in the context of improving the therapeutic utility of γδ T-cells in cancer management. A powerful approach that can be used to identify key transcriptomic regulators is trajectory inference (TI). In TI, cells are arranged in pseudo-time, which corresponds to stages along the development pathway of cells. A central assumption of TI is that cells with similar transcriptomes are close in developmental time. Various approaches for TI have been developed. The choice of method is highly dependent on prior knowledge of the differentiation trajectory, as the choice of the most appropriate tool for TI is dependent on trajectory topology (39).

## From single cell γδ T-cell characterization to cancer management

There is no doubt a growing interest in the use of γδ T-cells in cancer immunotherapy. Improved understanding of γδ TILs can provide mechanistic insights that enable better product manufacturing. This is demonstrated by Harmon and colleagues, who showed that exploiting metabolic differences between effector populations led to the generation of more a potent expanded cell products with higher cytotoxicity against CRC cell models (26). Additionally, integrative analysis of γδ T-cells with clinicopathological correlates is crucial to inform patient selection for γδ T-cell based therapies. Expanding our understanding of the biology of this unique cell population is critical in unlocking its potential as a novel therapeutic modality.

## Author contributions

JN: Writing – original draft, Writing – review & editing, Funding acquisition. AC: Writing – original draft, Writing – review & editing, Supervision, Funding acquisition.
